# Case report: Successful allogeneic stem cell transplantation in a child with novel GATA2 defect associated B-cell acute lymphoblastic leukemia

**DOI:** 10.3389/fimmu.2022.928529

**Published:** 2022-08-02

**Authors:** Edyta Heropolitańska-Pliszka, Barbara Piątosa, Anna Szmydki-Baran, Karolina Kuczborska, Karolina Miarka-Walczyk, Agata Pastorczak, Wojciech Młynarski, Łukasz Sędek, Tomasz Szczepański, Marek Ussowicz

**Affiliations:** ^1^ Department of Immunology, Children’s Memorial Health Institute, Warsaw, Poland; ^2^ Histocompatibility Laboratory, Children’s Memorial Health Institute, Warsaw, Poland; ^3^ Department of Oncology, Pediatric Hematology, Transplantology, and Pediatrics, Children’s Hospital, Medical University of Warsaw, Warsaw, Poland; ^4^ Department of Pediatrics, Nutrition and Metabolic Disorders, Children’s Memorial Health Institute, Warsaw, Poland; ^5^ Department of Pediatrics, Hematology and Oncology, Medical University of Lodz, Lodz, Poland; ^6^ Department of Microbiology and Immunology, Zabrze, Medical University of Silesia, Katowice, Poland; ^7^ Department of Pediatric Hematology and Oncology, Zabrze, Medical University of Silesia, Katowice, Poland; ^8^ Department and Clinic of Pediatric Oncology, Haematology and Bone Marrow Transplantation, Wroclaw Medical University, Wroclaw, Poland

**Keywords:** GATA2, acute lymphoblastic leukemia, immunodeficiency, hematopoietic stem cell transplantation, treosulfan

## Abstract

GATA-binding protein 2 (*GATA2*) is a transcription factor responsible for the regulation of blood cell proliferation, differentiation, and maintenance in hematopoietic stem cells. Here, we describe successful bone marrow transplantation in a carrier of a novel GATA2 pathogenic variant who was diagnosed with immunodeficiency a few years after completion of B-cell precursor acute lymphoblastic leukemia (BCP-ALL) treatment. At the age of 4 years, the patient was diagnosed with and treated for BCP-ALL. Antileukemic therapy was complicated by pulmonary cryptococcosis. Two years after completion of the maintenance therapy, the child was consulted by an immunologist because of recurrent respiratory tract infections and an episode of sepsis. Flow cytometry revealed deep monocytopenia, lymphopenia, absence of B lymphocytes, considerably reduced NK cells, poor thymic T lymphocyte production, minor defects in T cell maturation, and absence of TCRγδ+ T cells. The presence of the likely pathogenic, heterozygous missense variant within exon 5 of *GATA2* (NM_032638.5: c.1047T>G, Cys349Trp) was identified in the proband and confirmed in the father of the patient, who underwent allogeneic hematopoietic stem cell transplantation (HSCT) from a matched unrelated donor due to myelodysplastic syndrome with excess blasts at the age of 22 years. An allogeneic hematopoietic stem cell transplantation with a reduced toxicity conditioning protocol was performed using a matched sibling donor. Pre-transplant conditioning included fludarabine (5 × 30 mg/m2), treosulfan (3 × 14 g/m2), and thiotepa (10 mg/kg). Complete donor chimerism was achieved on post-transplant day 17. During the 12 months of the posttransplant observation period, she remained free from symptoms of acute or chronic graft-versus-host disease, and immunosuppressive treatment was therefore stopped. This is the second reported case of BCP-ALL in a patient with *GATA2* deficiency, and the first successfully treated with a reduced-toxicity conditioning HSCT protocol. The co-occurrence of lymphoid malignancies and primary immunodeficiencies points to the role of genetic counseling and family screening for possible cancer predisposition syndromes prior to the selection of related HSCT donors.

## Introduction

GATA-binding protein 2 (*GATA2*) is a transcription factor responsible for the regulation of blood cell proliferation and differentiation as well as the maintenance of hematopoietic stem cells (1, 2). Germline mutations that cause *GATA2* haploinsufficiency can lead to a wide spectrum of clinical symptoms. The main features include cytopenia, which can affect several cell lineages, including B cells, natural killer (NK) cells, CD4+ T cells, and monocytes. Impaired multi-lineage hematopoiesis may lead to clonal selection and evolution into myeloid neoplasms. *GATA2* induced immunodeficiency is associated with an increased risk of atypical mycobacterial, herpes virus infection, and fungal disease (3–6). Moreover, about half of the patients develop pulmonary complications, mainly pulmonary alveolar proteinosis (PAP), caused by the abnormal accumulation of surfactant, which worsens gas exchange and can lead to pulmonary arterial hypertension (7). Other complications include lymphedema, chronic warts that are irresponsive to treatment, an increased risk of skin cancers, hypothyroidism, and hearing impairment (8). *GATA2* deficiency is an inherited autosomal dominant trait or caused due to *de novo* mutations and is characterized by high penetrance (approximately 90% by the age of 60 years) (4, 8).

Although the influence of *GATA2* deficiency on the development of myeloid neoplasms is well documented, B-cell precursor acute lymphoblastic leukemia (BCP-ALL) has been reported in only one patient with monocytopenia and mycobacterial infection (MonoMAC) syndrome (9, 10). Therefore, we describe the phenotype of GATA2 deficiency during the first year of life in carriers of the novel familial *GATA2* mutation who developed immunodeficiency after ALL treatment. This case illustrates the importance of collecting the family history of children diagnosed with ALL and immunological testing of ALL patients who show recurrent infections a few years after the completion of oncological treatment.

## Methods

Peripheral blood sample was immunophenotyped by multicolor flow cytometry and fluorochrome-conjugated mouse monoclonal antibodies. The BD Multitest TM 6-color TBNK with Trucount tubes (Becton Dickinson, cat no. 337166) (CD3-FITC (clone SK7), CD16-PE (clone B73.1), CD56-PE (clone NCAM16.2), CD45-PerCP-Cy5.5 (clone 2D1), CD4-PE-Cy7 (clone SK3), CD19-APC (clone SJ25C1), CD8-APC-Cy7 (clone SK1)) was used as prescribed by the manufacturer to evaluate basic lymphocyte subsets. Maturation of the T helper and T suppressor/cytotoxic lymphocyte subsets was based on the differential expression of CD3PerCP (clone SK7, Becton Dickinson cat no. 345766), CD4-APC (clone SK3, Becton Dickinson cat no. 345771), CD8-APC-Cy7 (clone SK1, BD Pharmingen, cat no. 557834, CD27-APC (clone L128, Becton Dickinson cat. no. 337169), CD31-PE (clone WM59, BD Pharmingen cat. No 555446), CD45RA-FITC (clone HI100, BD Pharmingen cat. no. 555488), and CD45RO-FITC (clone UCHL1, BD Pharmingen cat. no 555492). Additionally, T regulatory cells and the distribution of TCRαβ and TCRγδ receptors were also evaluated (CD3-PerCP (clone SK7, Becton Dickinson cat no. 345766) CD4-APC (clone SK3, Becton Dickinson cat no 345771), CD25-FITC (clone 2A3, Becton Dickinson cat. No 340907), CD127-PE (clone HIL-7R-M21, BD Pharmingen, cat no. 557938), TCRαβ-FITC (clone WT31, BD Pharmingen 333140), and TCRγδ-PE (clone 11F2, Becton Dickinson, cat no. 333141). Briefly, 50 μl of the full blood sample was incubated with monoclonal antibodies for 15 min at room temperature. Erythrocytes were lysed for 15 min with a BD Lysing Solution. The samples were then washed with PBS+0.1% sodium azide. At least 2000 cells from the analyzed gate were acquired using an appropriately calibrated FACS Canto II flow cytometer (Becton Dickinson). The results were analyzed using the BD FACS Diva v.8 software (Becton Dickinson).

Bone marrow sample (BM) was subjected to flow cytometric immunophenotyping using a 4-tube, 8-color panel of fluorochrome-conjugated monoclonal antibodies against lymphoid and myeloid antigens (CD19-PE-Cy7, CD10-APC, CD22-PE, CD45-V500, CD34-PerCP-Cy5.5, CD38-APC-H7, CD117-PE, CD33-PE-Cy7, CD13-PE, CD15-FITC, CD11b-APC, CD64-PE, CD36-APC, CD4-APC, CD14-APC-H7 – Becton Dickinson, San Jose, CA, USA; HLADR-Pacific Blue, CD16-Pacific Blue, CD20-PacificBlue – Biolegend, San Diego, CA, USA; CD56-PE-Cy7 – Beckman Coulter, Brea, CA, USA). The samples were incubated with monoclonal antibodies for 15 min at room temperature. Erythrocytes were lysed by incubation for 10 min with an appropriately diluted BD Lysing Solution (Becton Dickinson). After erythrocyte lysis, the samples were washed with Cell Wash solution (Becton Dickinson) and analyzed using a FACS Canto II flow cytometer (Becton Dickinson). 300,000 cells per tube were recorded. The Infinicyt software was used for data analysis (Cytognos, Salamanca, Spain).

### DNA isolation

Genomic DNA was extracted from peripheral blood samples using the QIAamp DNA Blood Mini Kit (QIAGEN, Hilden, Germany) or from buccal swabs using the Sherlock AX Kit (A&A Biotechnology, Poland). The concentration and quality of the isolates were determined by ultraviolet spectrophotometry (NanoDrop 8000, Thermo Scientific, Waltham, USA).

### Direct sequencing

The presence of a likely pathogenic missense variant within exon 5 of *GATA2* (NM_032638.5: c.1047T>G, Cys349Trp) was identified by direct sequencing. Standard PCR conditions were applied with primers specifically designed to cover the *GATA2* coding region using NetPrimer software (**Supplementary Table 1**). Products were sequenced on an ABI3130 4-capillary sequencer (Thermo Fisher Scientific, Waltham, MA, USA), and the results were analyzed using Sequencher v. 5.0 (Gene Codes, Ann Arbor, USA).

## Case report

**Figure 1:**
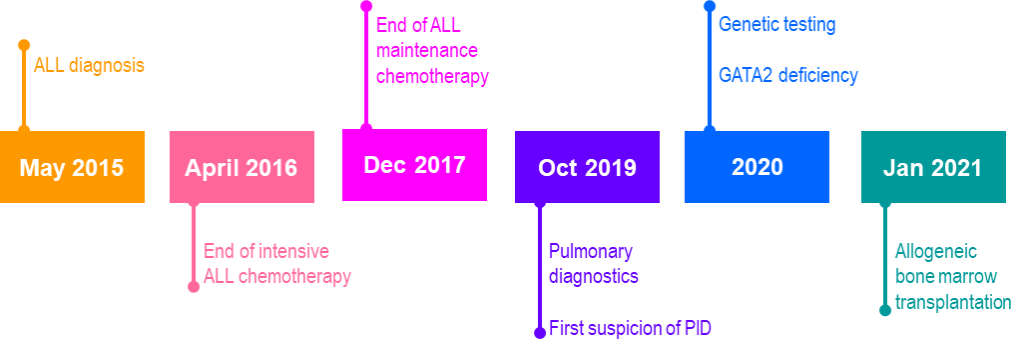
Case report timeline. ALL, acute lymphoblastic leukemia; PID, primary immunodeficiency.

A 4-year-old girl was referred to the oncology department for diagnosis and treatment of leukopenia, neutropenia, and anemia. The timeline of case report is shown in **Figure 1**. In the past, she had experienced several episodes of bronchitis and pneumonia. Leukopenia and anemia were discovered accidentally when she was treated with bilateral tympanostomy due to recurrent otitis media. A complete blood count with differential revealed profound neutropenia, thrombocytopenia, and lymphocytosis. Clinically, the patient presented with progressive weakness, and multiple sites of subcutaneous hemorrhage and cervical lymphadenopathy without hepatosplenomegaly were observed. Due to suspected malignancy the patient underwent hematological work-up with BM biopsy.

**Figure 2:**
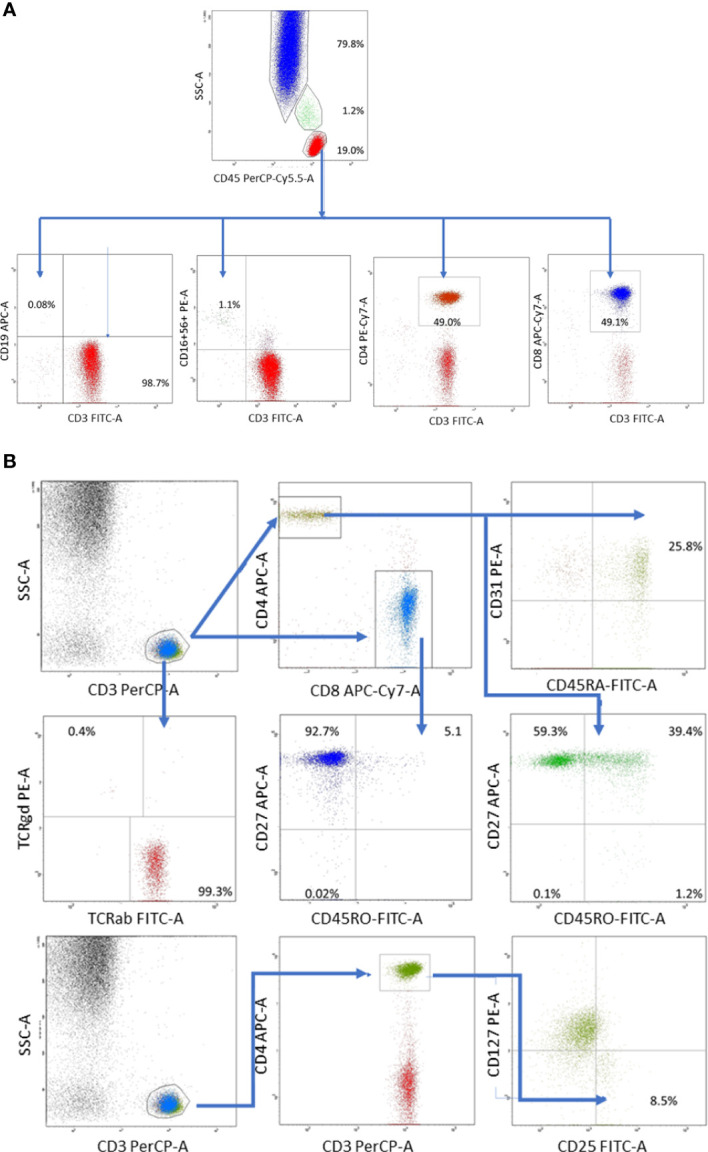
**(A)** Distribution of whole blood leukocyte subsets was based on differential expression of CD45 and side scatter characteristics: lymphocytes (low scatter and high CD45 expression), monocytes (medium side scatter and CD45 expression), and polymorphonuclears (high scatter and low CD45 expression) composed 19.0, 1.2% and CD79.8% of whole blood leukocytes, respectively. T lymphocytes were defined as CD3+CD19- cells, B lymphocytes as CD19+CD3- cells and composed 98.7% and 0.08% of total lymphocyte population, respectively. NK cells defined as CD3-CD16.CD56+ cells composed 1.1% of whole lymphocyte population.**(B)** T lymphocyte gate was set up based on high CD3 expression and low side scatter. Within T cell gate two subsets were identified: T helper (CD3+CD4+) and T suppressor (CD3+CD8+) cells. Recent thymic emigrants were identified as CD31+CD45RA+, while regulatory T cells as CD25+CD127- within T helper cell population. Remaining T lymphocyte maturation stages: naïve (CD27+CD45RO-), memory (CD27+CD45RO+), effector memory (CD27-CD45RO+) and effector (CD27-CD45RO-) were defined in the similar way within T helper (CD3+CD4+) and T suppressor (CD3+CD8+) gates. Distribution of T cell receptor variants was defined based on TCRαβ and TCRγδ expression within T cell gate).

At referral, lymphoblasts expressing CD34/CD9/CD10/CD19/CD20/CD22/CD24/CD33/CD38/CD66c/CD79a/CD81/TdT comprised 85% of the nucleated cells in the BM but were not found in the peripheral blood of the patient. Acute lymphoblastic leukemia of B-cell progenitor origin was diagnosed based on the results of 8-color flow cytometry performed with a standard Euroflow panel of fluorochrome-conjugated monoclonal antibodies (11). The patient responded well to the treatment according to the ALL IC-BFM 2009 protocol and showed 1.56% blast cells at day +15 and 0.08% blast cells at day +33 in the BM (12). Despite adequate trimethoprim prophylaxis, the patient developed symptoms resembling an upper respiratory tract infection. Although she received aggressive treatment with meropenem, vancomycin, amphothericin B, teikoplanin, ciprofloxacin, and clarithromycin, she developed an abscess in her right lung, which was positive for *Cryptococcus* antigen. Although mercaptopurin hypersensitivity was suspected because the patient demonstrated long periods of BM aplasia; however, thiopurin S-methyltransferase deficiency was excluded. Inflammatory lesions caused by *Streptococcus pneumoniae* MLSB were identified during bronchoscopy and effectively treated with antibiotics and antifungal agents accompanying long-term oncologic treatment. Pneumonia, probably of atypical bacterial origin, and reactivation of enteric *Clostridium* infection were also observed. Due to infectious complications, intensive phase of ALL chemotherapy was extended from 6 to 11 months. Consolidation and maintenance treatment were continued for 13 months, leading to mild leukopenia and neutropenia with normal monocyte counts after the completion of 32 months of chemotherapy. After the end of 2 years of oncologic treatment, the patient was referred by an outpatient immunologist for recurrent respiratory tract infections and a recent episode of sepsis. Fatigue, poor wound healing, and ecchymosis were also observed. Flow cytometry revealed deep monocytopenia, lymphopenia, absence of B lymphocytes, significantly reduced NK cells, poor thymic T lymphocyte production, minor defects in T cell maturation, and absence of TCRγδ+ T-cells (**Figure 2**). The values of the lymphocyte subpopulations with age-adjusted normal ranges are shown in **Table 1**.

**Table 1:** Results of peripheral blood lymphocyte subpopulations.

Parameter:			Absolute counts	Normal range (age 5 y - 10 y)	
		%	(cells/ul)	(%)	(cells/ul)
Lymphocytes		19.0↓		29.6-49.8	
Monocytes		1.2↓		6.1-12.5	
Polymorphonuclears		79.8↑		41.6-64.1	
Lymphocytes			787↓		1700-3600
T	CD3+	98.7↑	777↓	52.4-77.9	1000-2600
T CD8+	CD3+CD8+	49.1↑	387	15.0-35.4	300-1000
T CD4+	CD3+CD4+	49.0	386↓	26.7-46.2	500-1500
NK cells	CD16+56+CD3-	1.1↓	9↓	6.2-29.8	140-690
B lymphocytes	CD19+	0.08↓	1↓	9.7-23.7	300-600
CD4:CD8		1.0		0.8-2.5	
Recent thymic emigrants	CD31+CD45RA+ (%CD3+CD4+)	25.8↓		>40	
Naïve T CD4+	CD27+CD45RO-(%CD3+CD4+)	59.3		55.6-75.8	
Memory T CD4+	CD27+CD45RO+ (%CD3+CD4+)	39.4↑		22.5-37.0	
Effector memory CD4+	CD27-CD45RO+ (%CD3+CD4+)	1.2↓		1.5-9.7	
Effector CD4+	CD27-CD45RA-(%CD3+CD4+)	0.1		0.1- 0.3	
Treg	CD25+CD127- (%CD4)	8.5↑		1.8-7.4	
Naïve T CD8+	CD27+CD45RO-(%CD3+CD8+)	92.7↑		57.0-83.7	
Memory T CD8+	CD27+CD45RO+ (%CD3+CD8+)	5.1↓		9.2-22.6	
Effector memory CD8+	CD27-CD45RO+ (%CD3+CD8+)	0.2↓		0,7-14.0	
Effector CD8+	CD27-CD45RA-(%CD3+CD8+)	1.93		0.9-17.9	
TCRαβ	TCRαβ+ (%CD3)	99.3↑		78.5-93.8	
TCRγδ	TCRγδ+ (%CD3)	0.4↓		6.0-21.4	

↑, above normal range; ↓, below normal range.

**Figure 3:**
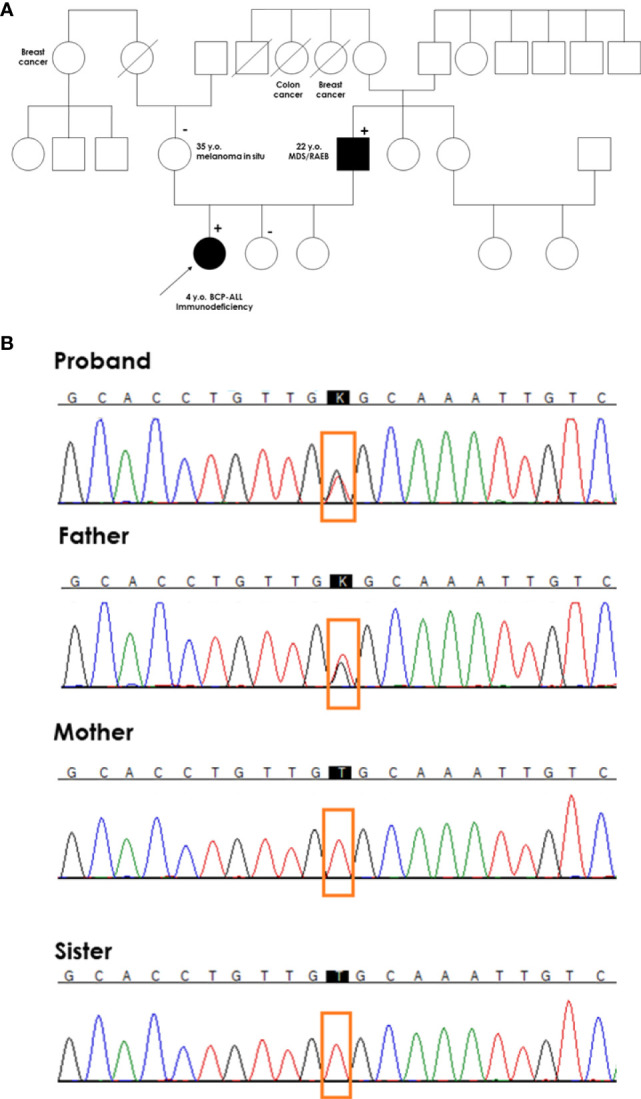
Genetic testing of the proband’s family. **(A)** A pedigree of the proband’s family. The proband with BCP-ALL had a germline mutation in exon 5 of the *GATA2* gene (c.1047T>G, Cys349Trp) inherited from her father with MDS. (+) denotes heterozygous mutation carrier in the germline; (-) denotes wild-type in the germline. A diagonal line through a symbol indicates that the person is deceased **(B)** Chromatograms of germline *GATA2* variant in the proband, and proband’s parents and sister.

The patient was consulted by a geneticist, and her family history was thoroughly examined. The father of the proband underwent allogeneic hematopoietic stem cell transplantation (HSCT) from a matched unrelated donor due to myelodysplastic syndrome (MDS) with excess blasts (EB) at the age of 22 years. The mother of the proband was diagnosed with melanoma *in situ* at the age of 35 years. Two paternal aunts died of breast and colon cancer, and one maternal aunt developed breast cancer (**Figure 3A**). The diagnosis of proband was based on immunophenotyping and cytogenetics, whereas the other oncological diagnoses in her family were based on self-reporting by the parents of the proband.

Because specific immunological abnormalities and BCP-ALL occurred in the proband whose father was treated for MDS and flow cytometry results were typical for *GATA2* deficiency, direct sequencing of the coding region of *GATA2* was performed. We identified a likely pathogenic heterozygous missense variant within exon 5 of *GATA2* (NM_032638.5; c.1047T > G, Cys349Trp). The mutation changed the structure of the 4 amino acids, forming a hinge between *GATA2* zinc finger domains ZF1 and ZF2. This mutation was also found in the symptomatic father, but was absent in healthy mother and sisters (**Figure 3B**).

**Figure 4:**
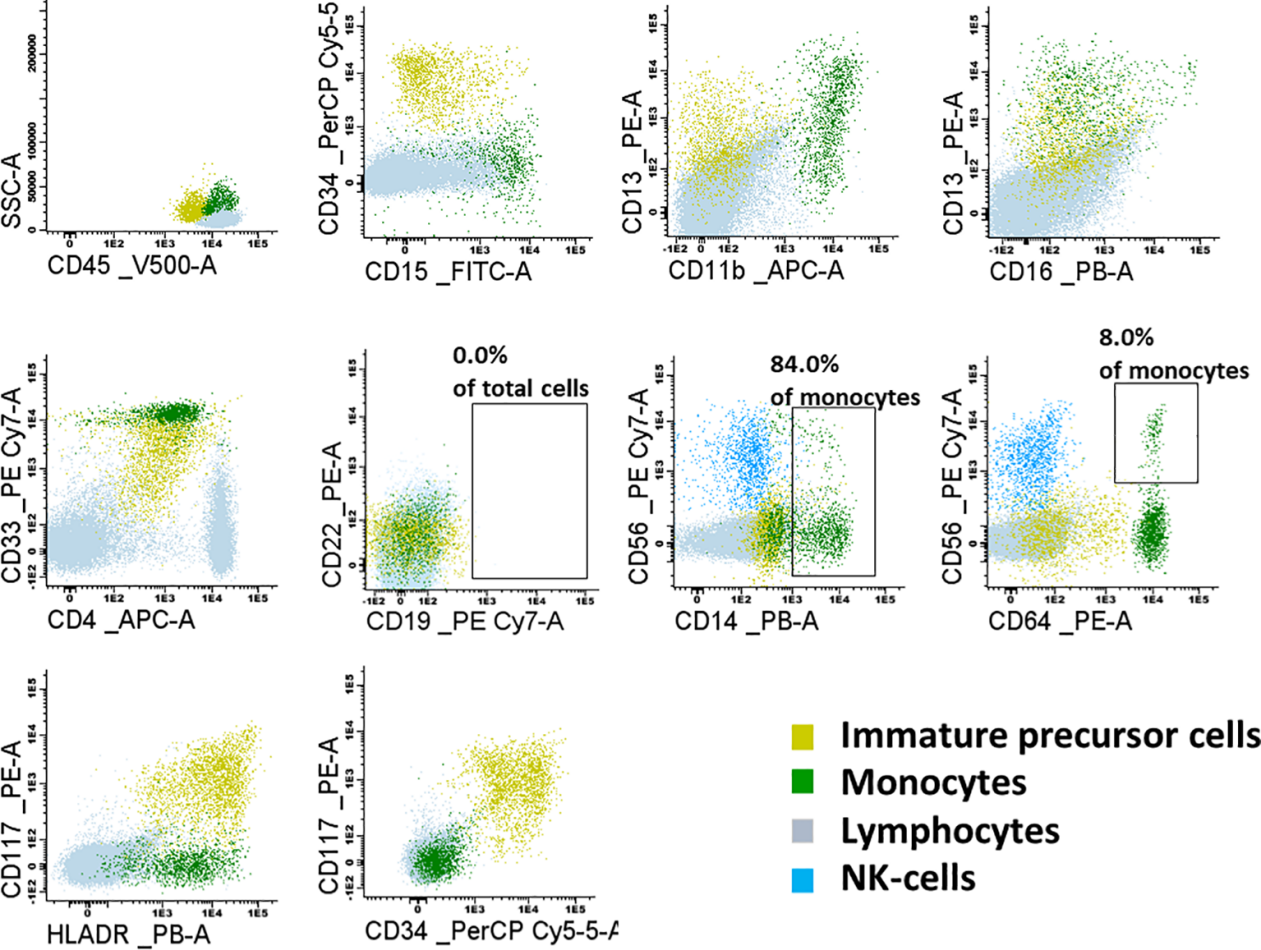
Flow cytometry dot-plots representing the lymphoid, monoid and immature precursor cell subsets in the examined bone marrow sample. Lymphoid subset was represented mostly by T-cells and NK-cells. No mature B-cells nor precursor B-cells were present. Monoid lineage was represented mainly by mature monocytes (84.0% of CD14+); 8% of monocytes were CD56+.

The child qualified for allogeneic HSCT from an HLA-matched sibling donor (older sister). Due to the constitutional defect in the family, a sister was tested for *GATA2* deficiency and confirmed to have a wild-type *GATA2* sequence. Before transplantation, the child underwent diagnostic BM biopsy, and the sample was analyzed with flow cytometry for minimal residual disease (MRD) to exclude the presence of leukemic cells and to investigate maturation abnormalities in the hematopoietic compartment. Bone marrow smear showed normal did not present morphologic abnormalities (**Supplementary Figure 1**), and all cell lineages were represented and their proportions were appropriate for age (**Supplementary Table 2**). BM MRD immunophenotyping confirmed BCP-ALL remission with a balanced distribution of myeloid cells (46.8% of total cells, mostly neutrophils at different stages of maturation) and erythroid cells (total of 41.4%). Lymphoid, myeloid, and immature precursor cell subsets comprised 8.4%, 0.49%, and 0.58% of the total cells, respectively (**Figure 4**). Lymphoid cell subsets were represented by T cells and NK cells (94.8% and 5.2% of total lymphocytes, respectively), while no mature B cells or precursor B cells were present. The myeloid lineage constituted 0.49% of total BM cells, mostly of the mature phenotype (CD14+, CD64++, CD33++, CD11b+, CD36+, CD13+, CD117-); 8% of monocytes exhibited expression of CD56.

After confirming complete remission, the patient was administered megatherapy. Pre-transplant conditioning included fludarabine (5 × 30 mg/m ^2^), treosulfan (3 × 14 g/m ^2^), and thiotepa (10 mg/kg). No serotherapy was administered, and posttransplant graft-versus-host disease (GvHD) prophylaxis consisted of methotrexate on posttransplant days 1, 3, and 6, along with cyclosporine A. The grafting material was obtained from bone marrow with a CD34+ cell dose of 1.27 x 10 ^6^ cells/kg of the recipient’s body weight. The posttransplant period was complicated by grade 4 mucositis and asymptomatic *S. haemolyticus* bacteremia treated with targeted antibiotic therapy (vancomycin). The girl showed neutrophil engraftment on post-transplant day +17, and mononuclear cell chimerism studies confirmed full donor engraftment. Until discharge on post-transplant day +40, the patient presented with nausea and drug swallowing problems that were not associated with demonstrable organic findings. During 12 months of posttransplant observation, the patient showed both BM recovery and immune reconstitution (**Supplementary Figure 2**), and did not manifest any infectious or non-infectious complications; in particular, she remained free from symptoms of acute or chronic graft-versus-host disease, and thus immunosuppressive treatment was successfully stopped.

## Discussion

*GATA2* was discovered in 2011 as an MDS/AML predisposition gene. Hahn et al. in their study found two different mutations in *GATA*2 in two families with a predisposition to MDS/AML (3). Further research has shown that the mutation spectrum is broad and includes missense, nonsense, frameshift, complex mutations, splice defects, and even whole-gene deletions (13). The molecular landscape of *GATA2* deficiency is more complicated than initially suggested. According to Kozyra et al. some *GATA2* synonymous mutations do not alter protein function or stability but can lead to splicing errors or late-stage RNA loss without splicing disruption (14). Nevertheless, new mutations are still being identified, as in the case of reported patient. Moreover, in contrast to adult mutation carriers, who manifest a complex GATA2 deficiency phenotype, initial clinical manifestation can be scarce and diagnosis challenging at a young age. Monocytopenia, deemed to be a hallmark of *GATA2* deficiency, may be absent at an early age or even an elevated monocyte count can be observed in these patients (15).

It has been postulated that *GATA*2 mutations are the most common germline defects that predispose to pediatric MDS, but importantly, they do not worsen the prognosis (15). However, the identification of such inherited predisposition genes is crucial for family screening, faster diagnosis, bone marrow transplant therapy, and selection of potential related donors (3).

Nonetheless, the diagnosis in our patient was difficult not only due to a new, so far unknown mutation, but also due to the reduced vigilance resulting from an atypical neoplasm. The influence of *GATA2* deficiency on AML/MDS development has been well described; however, the relationship between mutations in this gene and lymphoid neoplasms has not been widely documented. In 2016, Koegel et al. were the first to describe a case of B-ALL in an 11-year-old girl who was later diagnosed with MonoMAC syndrome and, therefore, with *GATA2* haploinsufficiency. After ALL relapse, she underwent HSCT but eventually died 3 months later due to neurologic injury resulting from leukemic brain infiltrates (10). Two years later, in 2018, Esparza et al. reported the first case of T-cell ALL in an 8-year-old girl with the *GATA2* mutation. Interestingly, this patient did not undergo HSCT because of her good clinical condition, with monthly immunoglobulin substitution and constant prophylaxis with azithromycin (9). In the same year, Donadieu et al. revealed one other case of T-cell ALL and one case of juvenile myelomonocytic leukemia among 79 surveyed patients with *GATA2* haploinsufficiency from French and Belgian populations (13). Our patient is the second ever reported patient with *GATA2* deficiency who developed BCP-ALL, but the first one showing long-term survival and with a familial history of *GATA2* myeloid malignancy. One of the most intriguing conundrums in the observed patients is the fact that the reported *GATA2* variant associated with leukemogenesis was not lineage specific. Neither the case described by us nor those presented by other investigators explains the relationship between *GATA2* haploinsufficiency and the development of lymphoblastic neoplasms. Several authors have attempted to find a deleterious *GATA2* mutation in familial and sporadic cases of lymphoblastic tumors (3, 16). Collin et al. suggested that the *GATA2* transcription factor plays not only an important role in early hematopoiesis, but it can also affect B cell differentiation (17). It would be interesting to investigate somatic aberrations present in the leukemic sample of our patient. This could potentially shed light on the acquisition of cooperative gene lesions that promote leukemic evolution. Although *GATA2* activation has been observed in BCP-ALL cells, the role of *GATA2* haploinsufficiency in leukemogenesis has not been addressed (18).

Cell subpopulation studies in the peripheral blood and BM showed maturation abnormalities in the patient. In patients who developed *GATA2*-related MDS, monocytosis was observed at the initial stage (19, 20). Loss of B cells and their precursors is the most constant feature of *GATA2* deficiency, especially in patients who develop MDS, when monocytopenia might be masked by progenitor cell expansion and total lymphocyte counts maintained by expanding memory T cells (21). In the reported case, we did not observe an abundance of CD8+ T cells expressing CD45RA (TEMRA phenotype) or loss of CD27, in contrast to an earlier report (8).

The curative role of HSCT in *GATA2* deficiency has been firmly established in patients with myeloid malignancies; however, in earlier cohorts, only a minority of *GATA2* deficient patients were referred for transplantation (13). Recent worldwide experience with patients having *GATA2* deficiency suggests the need for HSCT in as many as 80% of these cases (22). The best timing of HSCT has not yet been established, because some patients with *GATA2* deficiency show long-term survival, and genetic reversal due to somatic rescue mutations has been reported (23). A reasonable recommendation is to perform HSCT before *GATA2* deficient patients develop malignancies or severe or recurrent infections that lead to organ failure (24). At the time of preparing this manuscript, there are no recommendations on the choice of conditioning protocol for *GATA2* deficiency. In children with myelodysplastic syndromes, HSCT outcomes were independent of *GATA2* germline mutations, but the cytogenetic profile and BM blast count were associated with different prognoses and megatherapy protocols (25). Hoffman et al. analyzed the outcomes of HSCT after myeloablative conditioning and reported an elevated risk of neurologic toxicities and post-HSCT thrombotic events in the *GATA2* cohort (26). However, these findings were not confirmed by Bortnick et al (25). In contrast, in a recently published paper, a busulfan-based myeloablative conditioning protocol was associated with typical complications and resulted in an 85.1% OS after 4 years (27). The conditioning protocol for our patient was chosen based on the fact that she was in complete remission, and BCP-ALL itself was not considered an unfavorable risk factor or an indication for HSCT alone. The leading indication for HSCT in this case was immunodeficiency, and the patient was prepared with a treosulfan-fludarabine-based reduced toxicity conditioning (RTC) regimen with the addition of thiotepa, similar to the ESID guidelines (28, 29). RTC protocols are associated with a lower incidence of late sequelae, such as endocrinopathy, which is important in children with long-term survival expectation (30).

In conclusion, we report a rare occurrence of BCP-ALL in *GATA2* deficiency patients successfully treated with a reduced toxicity conditioning HSCT protocol. The medical history of patient draws attention to the possible co-occurrence of lymphoid malignancies with primary immunodeficiencies and cancer predisposition syndromes. Genetic diagnosis of inherited bone marrow dysfunction was essential for the appropriate treatment of the patient and screening of her family.

## Data availability statement

The original contributions presented in the study are included in the article/**Supplementary Material**. Further inquiries can be directed to Marek Ussowicz, marek.ussowicz@umw.edu.pl.

## Ethics statement

Written informed consent was obtained from the individual(s) and/or minor(s)’ legal guardian/next of kin for the publication of any potentially identifiable images or data included in this article.

## Author contributions

Concept, data collection, analysis, manuscript preparation and acceptance: EH-P, MU. Data collection, molecular studies, manuscript acceptance: KM-W, AP, WM. Data collection, immunological studies, manuscript acceptance: BP, ŁS, TS. Data collection, patient care, manuscript acceptance: AS-B, KK. All authors have read and approved the final manuscript. All authors contributed to the article and approved the submitted version.

## Funding

Source of support- Wroclaw Medical University statutory grant SUBZ.C200.22.067.

## Acknowledgements

We would like to express our special thanks of gratitude to prof. Wojciech Feleszko for patient’s referral.

## Conflict of interest

The authors declare that the research was conducted in the absence of any commercial or financial relationships that could be construed as a potential conflict of interest.

## Publisher’s note

All claims expressed in this article are solely those of the authors and do not necessarily represent those of their affiliated organizations, or those of the publisher, the editors and the reviewers. Any product that may be evaluated in this article, or claim that may be made by its manufacturer, is not guaranteed or endorsed by the publisher.

## References

[1] Tsai FY Orkin SH . Transcription factor GATA-2 is required for proliferation/survival of early hematopoietic cells and mast cell formation, but not for erythroid and myeloid terminal differentiation. Blood (1997) 89(10):3636–43. doi: 10.1182/blood.V89.10.3636 9160668

[2] Orkin SH . GATA-binding transcription factors in hematopoietic cells. Blood (1992) 80(3):575–81. doi: 10.1182/blood.V80.3.575.575 1638017

[3] Hahn CN Chong C-E Carmichael CL Wilkins EJ Brautigan PJ Li X-C . Heritable GATA2 mutations associated with familial myelodysplastic syndrome and acute myeloid leukemia. Nat Genet (2011) 43(10):1012–7. doi: 10.1038/ng.913 PMC3184204 21892162

[4] Hsu AP Sampaio EP Khan J Calvo KR Lemieux JE Patel SY . Mutations in GATA2 a re associated with the autosomal dominant and sporadic monocytopenia and mycobacterial infection (MonoMAC) syndrome. Blood (2011) 118(10):2653–5. doi: 10.1182/blood-2011-05-356352 PMC3172785 21670465

[5] Dickinson RE Milne P Jardine L Zandi S Swierczek SI McGovern N . The evolution of cellular deficiency in GATA2 mutation. Blood (2014) 123(6):863–74. doi: 10.1182/blood-2013-07-517151 PMC3916878 24345756

[6] Mir MA Kochuparambil ST Abraham RS Rodriguez V Howard M Hsu AP . Spectrum of myeloid neoplasms and immune deficiency associated with germline GATA 2 mutations. Cancer Med (2015) 4(4):490–9. doi: 10.1002/cam4.384 PMC4402062 25619630

[7] Ishii H Tazawa R Kaneko C Saraya T Inoue Y Hamano E . Clinical features of secondary pulmonary alveolar proteinosis: pre-mortem cases in Japan. Eur Respir J (2011) 37(2):465–8. doi: 10.1183/09031936.00092910 21282812

[8] Spinner MA Sanchez LA Hsu AP Shaw PA Zerbe CS Calvo KR . GATA2 deficiency: a protean disorder of hematopoiesis, lymphatics, and immunity. Blood (2014) 123(6):809–21. doi: 10.1182/blood-2013-07-515528 PMC3916876 24227816

[9] Esparza O Xavier AC Atkinson TP Hill BC Whelan K . A unique phenotype of T-cell acute lymphoblastic leukemia in a patient with GATA2 haploinsufficiency. Pediatr Blood Cancer. (2019) 66(6):e27649. doi: 10.1002/pbc.27649 30802360

[10] Koegel AK Hofmann I Moffitt K Degar B Duncan C Tubman VN . Acute lymphoblastic leukemia in a patient with MonoMAC syndrome/GATA2 haploinsufficiency. Pediatr Blood Cancer. (2016) 63(10):1844–7. doi: 10.1002/pbc.26084 27232273

[11] van Dongen JJM Lhermitte L Böttcher S Almeida J van der Velden VHJ Flores-Montero J . EuroFlow antibody panels for standardized n-dimensional flow cytometric immunophenotyping of normal, reactive and malignant leukocytes. Leukemia (2012) 26(9):1908–75. doi: 10.1038/leu.2012.120 PMC3437410 22552007

[12] ALL IC-BFM 2009 a randomized trial of the I-BFM-SG for the management of childhood non-b acute lymphoblastic leukemia. final version of therapy protocol from august-14-2009 (2009). Available at: https://www.bialaczka.org/wp-content/uploads/2016/10/ALLIC_BFM_2009.pdf.

[13] Donadieu J Lamant M Fieschi C de Fontbrune FS Caye A Ouachee M . Natural history of GATA2 deficiency in a survey of 79 French and Belgian patients. Haematologica (2018) 103(8):1278–87. doi: 10.3324/haematol.2017.181909 PMC6068047 29724903

[14] Kozyra EJ Pastor VB Lefkopoulos S Sahoo SS Busch H Voss RK . Synonymous GATA2 mutations result in selective loss of mutated RNA and are common in patients with GATA2 deficiency. Leukemia (2020) 34(10):2673–87. doi: 10.1038/s41375-020-0899-5 PMC7515837 32555368

[15] Wlodarski MW Hirabayashi S Pastor V Starý J Hasle H Masetti R . Prevalence, clinical characteristics, and prognosis of GATA2-related myelodysplastic syndromes in children and adolescents. Blood (2016) 127(11):1387–97. doi: 10.1182/blood-2015-09-669937 26702063

[16] Hamadou WS Mani R Besbes S Bourdon V Youssef Ben Eisinger Y F . GATA2 gene analysis in several forms of hematological malignancies including familial aggregations. Ann Hematol (2017) 96(10):1635–9. doi: 10.1007/s00277-017-3076-9 28752392

[17] Collin M Dickinson R Bigley V . Haematopoietic and immune defects associated with GATA2 mutation. Br J Haematol (2015) 169(2):173–87. doi: 10.1111/bjh.13317 PMC4409096 25707267

[18] Wang H Cui B Sun H Zhang F Rao J Wang R . Aberrant GATA2 activation in pediatric b-cell acute lymphoblastic leukemia. Front Pediatr (2022) 9:795529. doi: 10.3389/fped.2021.795529 35087776 PMC8787225

[19] Wlodarski MW Collin M Horwitz MS . GATA2 deficiency and related myeloid neoplasms. Semin Hematol (2017) 54(2):81–6. doi: 10.1053/j.seminhematol.2017.05.002 PMC5650112 28637621

[20] Cada M Lara-Corrales I Dror Y Feanny S Hong-Diep Kim V Grunebaum E . Monocytosis in a patient with a novel GATA2 mutation. LymphoSign J (2015) 2(2):85–90. doi: 10.14785/lpsn-2014-0022

[21] Novakova M Aliova M Sukova M Wlodarski M Janda A Fro kova E . Loss of b cells and their precursors is the most constant feature of GATA-2 deficiency in childhood myelodysplastic syndrome. Haematologica (2016) 101(6):707–16. doi: 10.3324/haematol.2015.137711 PMC5013954 27013649

[22] Jørgensen SF Buechner J Myhre AE Galteland E Spetalen S Kulseth MA . A nationwide study of GATA2 deficiency in Norway–the majority of patients have undergone allo-HSCT. J Clin Immunol (2022) 42(2):404–20. doi: 10.1007/s10875-021-01189-y PMC8664000 34893945

[23] Catto LFB Borges G Pinto AL Clé D V. Chahud F Santana BA . Somatic genetic rescue in hematopoietic cells in GATA2 deficiency. Blood (2020) 136(8):1002–5. doi: 10.1182/blood.2020005538 32556109

[24] Baliakas P Tesi B Wartiovaara-Kautto U Stray-Pedersen A Friis LS Dybedal I . Nordic Guidelines for germline predisposition to myeloid neoplasms in adults: Recommendations for genetic diagnosis, clinical management and follow-up. HemaSphere (2019) 3(6):e321. doi: 10.1097/HS9.0000000000000321 31976490 PMC6924562

[25] Bortnick R Wlodarski M de Haas V De Moerloose B Dworzak M Hasle H . Hematopoietic stem cell transplantation in children and adolescents with GATA2-related myelodysplastic syndrome. Bone Marrow Transplant. (2021) 56(11):2732–41. doi: 10.1038/s41409-021-01374-y PMC8563415 34244664

[26] Hofmann I Avagyan S Stetson A Guo D Al-Sayegh H London WB . Comparison of outcomes of myeloablative allogeneic stem cell transplantation for pediatric patients with bone marrow failure, myelodysplastic syndrome and acute myeloid leukemia with and without germline GATA2 mutations. Biol Blood Marrow Transplant. (2020) 26(6):1124–30. doi: 10.1016/j.bbmt.2020.02.015 PMC7276093 32088370

[27] Nichols-Vinueza DX Parta M Shah NN Cuellar‐Rodriguez JM Bauer TR West RR . Donor source and post-transplantation cyclophosphamide influence outcome in allogeneic stem cell transplantation for GATA2 deficiency. Br J Haematol (2022) 196(1):169–78. doi: 10.1111/bjh.17840 PMC8702451 34580862

[28] Ussowicz M . Treosulfan in combination with fludarabine as part of conditioning treatment prior to allogeneic hematopoietic stem cell transplantation. Drugs Today (2020) 56(6):389. doi: 10.1358/dot.2020.56.6.3135200 32525137

[29] ESID EBMT HSCT guidelines 2017 (2017). Available at: https://esid.org/layout/set/print/content/download/15402/422689/file/ESID EBMT HSCT Guidelines 2017.pdf.

[30] Faraci M Diesch T Labopin M Dalissier A Lankester A Gennery A . Gonadal function after busulfan compared with treosulfan in children and adolescents undergoing allogeneic hematopoietic stem cell transplant. Biol Blood Marrow Transplant. (2019) 25(9):1786–91. doi: 10.1016/j.bbmt.2019.05.005 31082473

