# Supplementary material for: Case report: Successful allogeneic stem cell transplantation in a child with novel GATA2 defect associated B-cell acute lymphoblastic leukemia
Source: Front Immunol. 2022 Aug 2;13:928529. doi: 10.3389/fimmu.2022.928529 (PMC9378963; doi:10.3389/fimmu.2022.928529)
Supplement: Supplementary file 1 [file DataSheet_1.pdf]

Supplementary file

Supplementary Table 1. Sequences of primers used for direct sequencing of *GATA-2* coding region.

Primer	Sequence
F1	5'-GTGCCCCCTCCCCCTTTCT-3'
R1	5'-CGAGACCCTAACCCCGCCA-3'
F2	5'-TGGGCTTCTTAGGCGTGCG-3'
R2	5'-CTCTCAACAAAGCACACCAAAGC-3'
F3	5'-GCGTGCGGGACACCTCGT-3'
R3	5'-CCCTCCTCCCCTCCCTCG-3'
F4	5'-ATCTCAATGTCTGTCAGGGGCG-3'
R4	5'-CAAATGCTCCCCTCTTCCACG-3'
F5	5'-ACTCCCTCCCGAGAACTTGCC-3'
R5	5'-CCCAGCAGCCCCCTCCC-3'
F6	5'-GTGAGATTTAGCCCTCCTTGACTGA-3'
R6	5'-TTCCCAAGCCAAGCCAAGC-3'
F7	5'-GAGGAATGTTGCTGGAGGAAGGA-3'
R7	5'-GCGGTGGGGAACATTCACAGTA-3'

Supplementary Table 2. Bone marrow smear before HSCT (at the age of 11 years).

Cell lineage	%
**A. Erythroblastic lineage**	**46 %**
proerytroblasts	0.25 %
basophilic erythroblasts	4.0 %
polychromatic erythroblasts	26.75 %
orthochromatic erythroblasts	13 %
megaloblasts	2 %
**B. Myeloid lineage**	**47 %**
myeloblasts	0.5 %
promyelocytes	4.0 %
neutrophilic myelocytes	9.5 %
eosinophilic myelocytes	0.75 %
neutrophilic metamyelocytes	7 %
eosinophilic metamyelocytes	1.25 %
band neutrophils	9 %
neutrophils	12.75 %
eosinophils	1.75 %
basophils	0.5 %
**C. Lymphoid lineage**	**6.5 %**
lymphocytes	6.5 %
**D. Monocytes**	**0.5 %**

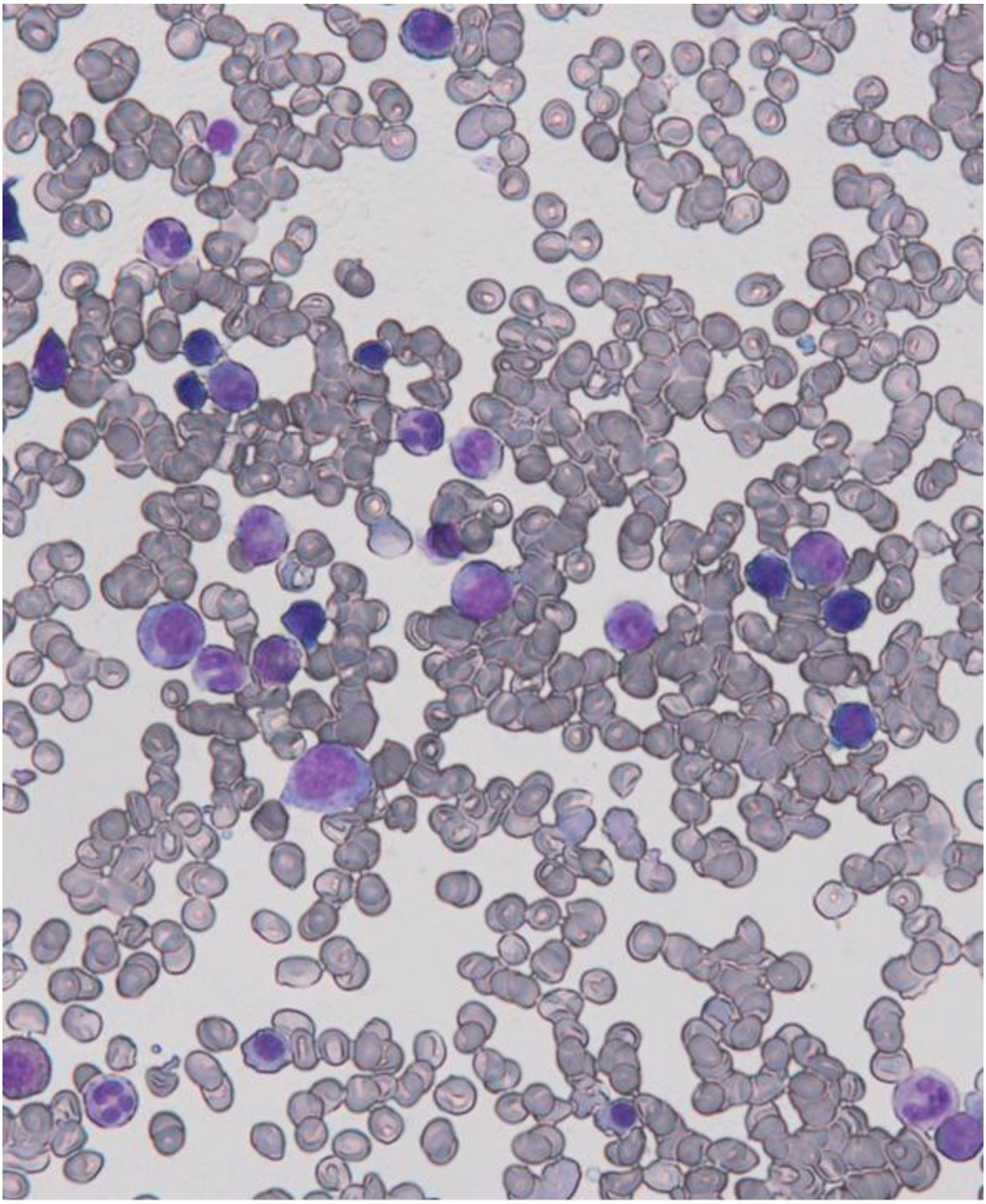

Supplementary figure 1. Low power view of a normocellular bone marrow aspirate from a patient with GATA2 defect (May-Grunwald-Giemsa stain).

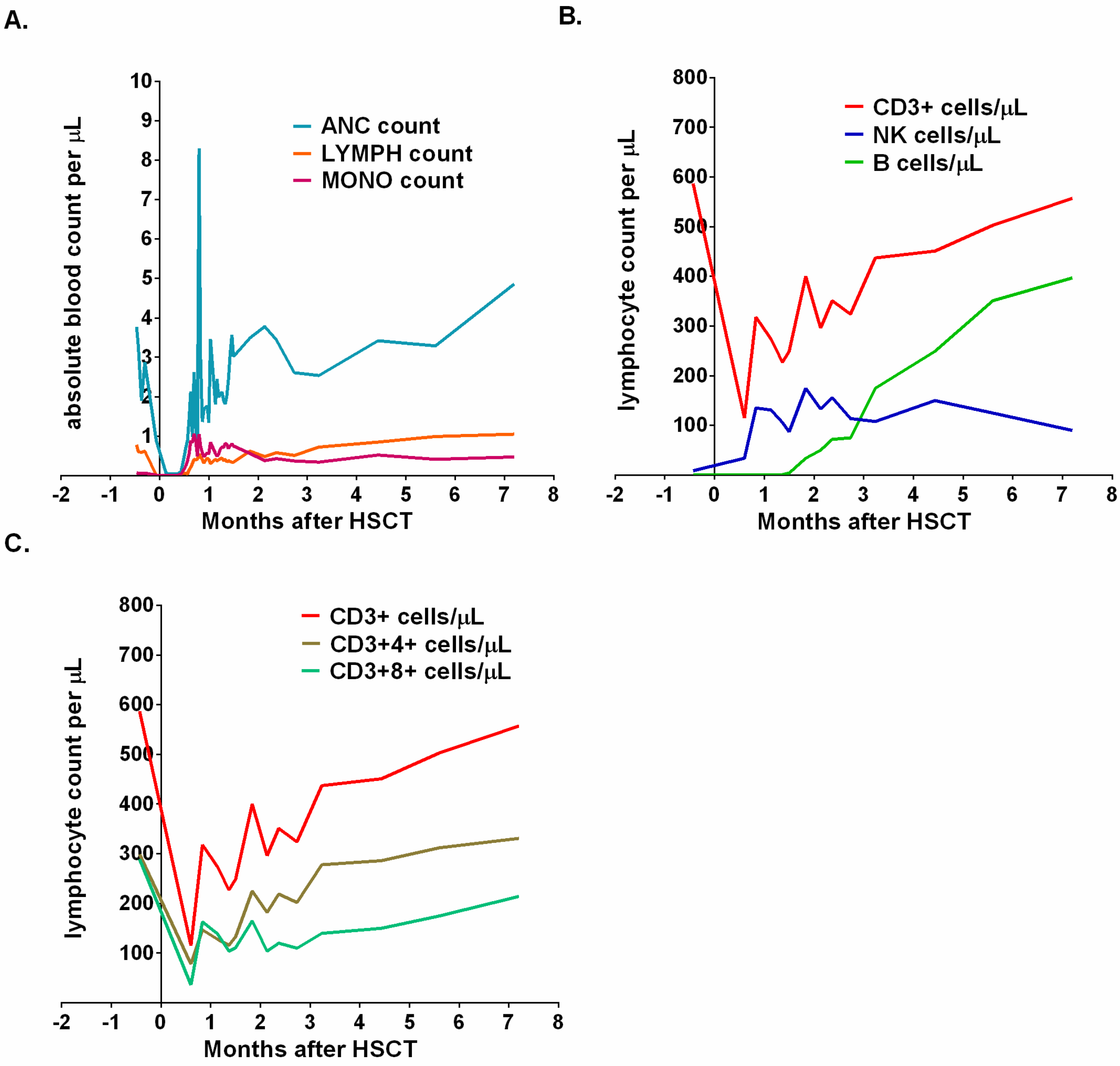

Supplementary figure 2. Absolute lymphocyte (LYMPH), monocyte (MONO) and neutrophil (ANC) counts in the peripheral blood (A). T, B lymphocyte and NK cell counts (B), and T CD3+4+ and CD3+8+ counts (C) after HSCT.
